# CRISPR correction of the Finnish ornithine delta-aminotransferase mutation restores metabolic homeostasis in iPSC from patients with gyrate atrophy

**DOI:** 10.1016/j.ymgmr.2022.100863

**Published:** 2022-04-01

**Authors:** Rocio Maldonado, Sami Jalil, Timo Keskinen, Anni I. Nieminen, Mervi E. Hyvönen, Risto Lapatto, Kirmo Wartiovaara

**Affiliations:** a Stem Cells and Metabolism Research Program, Faculty of Medicine, University of Helsinki, Helsinki, Finland; b Metabolomics Unit, Institute for Molecular Medicine Finland, University of Helsinki, Helsinki, Finland; c Children's Hospital, University of Helsinki and Helsinki University Hospital, Helsinki, Finland; d Clinical Genetics, Helsinki University Hospital, Helsinki, Finland

**Keywords:** CRISPR/Cas9, gene editing, gyrate atrophy, HOGA OAT

## Abstract

Hyperornithinemia with gyrate atrophy of the choroid and retina (HOGA) is a severe recessive inherited disease, causing muscular degeneration and retinochoroidal atrophy that progresses to blindness. HOGA arises from mutations in the ornithine aminotransferase *(OAT)* gene, and nearly one-third of the known patients worldwide are homozygous for the Finnish founder mutation OAT c.1205 T > C p.(Leu402Pro). We have corrected this loss-of-function *OAT* mutation in patient-derived induced pluripotent stem cells (iPSCs) using CRISPR/Cas9. The correction restored OAT expression in stem cells and normalized the elevated ornithine levels in cell lysates and cell media. These results show an efficient recovery of OAT function in iPSC, encouraging the possibility of autologous cell therapy for the HOGA disease.

## 1 Introduction

Hyperornithinemia with gyrate atrophy of choroid and retina (HOGA) is a severe autosomal recessively inherited metabolic disease, causing progressive visual degeneration [1,2] without curative treatment to date. Clinical manifestations include retinochoroidal atrophy, early cataract formation and type-II muscle fiber degeneration. These symptoms start in childhood, progressing to blindness and loss of type-II muscle fibers in adulthood [3,4].

HOGA arises from deleterious mutations in the ornithine aminotransferase (*OAT*) gene that lead to loss of enzymatic activity and ensuing high ornithine concentrations [[5], [6], [7]]. A strict Arg-restrictive diet reduces plasma ornithine concentration and slows down disease progression [8], suggesting ornithine toxicity in certain cell types.

The prevalence of HOGA is particularly high in Finland, where the overall carrier frequency nears 1:151 (*versus* the global 1;833) [9], with more than 90% of the patients presenting with the pathogenic Finnish mutation OAT c.1205 T > C p.(Leu402Pro) in homozygosity (hereafter HOGA_Fin_ or *c.1205* *T* *>* *C,* [9,10]).

A mouse model with OAT deficiency has been constructed [11]. When untreated, these animals recapitulate the progression of the human retinal disease. Moreover, the dietary treatment since weaning prevents retinal degeneration in the murine eye [12]. For human patients, however, this strict diet is difficult to accomplish, and the current treatments have been shown to delay, but not to prevent, the degeneration of the retina and choroid [[13], [14], [15]]. Hence, the need for an effective long-term treatment remains unmet.

Gene-editing tools offer novel therapeutic opportunities for many genetic diseases [16]. For ophthalmological pathologies, CRISPR/Cas9 systems have proven to efficiently correct specific disease mutations, either by homology-directed repair (HDR) or by single base editing [17,18]. These successful precedents have placed these technologies under the spotlight for the development of advanced therapies, some already in clinical trials [19].

Here, we have generated iPSC lines from two HOGA_Fin_ patients, and subsequently corrected the pathogenic mutation using Cas9-induced HDR. These corrected isogenic iPSC lines served to establish and characterize the first patient-derived iPSC model of HOGA_Fin_. We observed a restoration of OAT enzyme production and function upon genetic correction of the mutation. The recovered aminotransferase activity lowered the elevated ornithine levels in cell cultures. This further normalized the concentrations of other metabolites in related pathways [[20], [21], [22]]. Additionally, the corrected iPSC lines were geno- and phenotypically normal, and we did not detect any off-target genetic modifications, suggesting an acceptable safety profile. As the pathomechanisms of the disease remain unclear and the current treatment does not halt disease progression, we hope that our model will facilitate more detailed studies on pathophysiology and help to develop new therapeutic alternatives.

## 2 Patients, material and methods

Additional materials and methods can be found in Supplementary data.

### 2.1 Patients

We collected skin biopsies from two non-related voluntary female patients (aged 18 and 30). The patients were diagnosed in their childhood and have been on arginine-restricted diet ever since. Creatine and lysine supplementation started later in life. Both are homozygous for the most common Finnish OAT mutation c.1205 T > C p.(Leu402Pro).

### 2.2 Ethical consent

The Coordinating Ethics Committee of the Helsinki and Uusimaa Hospital District approved the generation of the induced pluripotent stem cell lines upon informed consent of the donors (Nro #HUS/2754/2019).

### 2.3 Fibroblast reprogramming

We transfected 1 × 10^6 human fibroblasts with CRISPR/Cas9 activators, as previously described [23]. Briefly, we detached the fibroblasts as single cells and washed them with PBS. Using the Neon transfection system (ThermoFisher; 1650 V, 10 ms, and 3 pulses), we electroporated 6 μg of plasmid mixture, (2 μg of dCas9 activator plasmid and 4 μg of guide plasmids) into the cells in a 100 μl transfection tip. Treated fibroblasts were plated on Matrigel -coated plates (Corning) in DMEM;10%FBS (ThermoFisher), later (d4) changed to a 1:1 mixture of DMEM;10%FBS and hES-medium (Gibco) supplemented with sodium butyrate (0.25 mM; Sigma).

iPSC colonies were picked manually (d15) and plated on Matrigel-coated wells in E8 medium. Media were changed every other day and cells were expanded up to passage 10. Thereafter, we used PCR targeting EBNA1 (Fw: 5’-ATCGTCAAAGCTGCACACAG −3′; Rv: 5’-CCCAGGAGTCCCAGTAGTCA −3′, Sigma) and OriP (Fw: 5’-TTCCACGAGGGTAGTGAACC -3′; Rv: 5’-TCGGGGGTGTTAGAGACAAC -3′, Sigma) to confirmed that the reprogramming plasmids had not integrated into the genome [24].

### 2.4 sgRNA and DNA donor template design

For genome editing of the disease mutation, we designed a suitable guide RNA using online tools (CRISPOR, [25]). We generated the sgRNA by incubating 5 min at 95 °C our gRNA (customized Alt-R CRISPR-Cas9 gRNA, iDT) with Alt-R® CRISPR-Cas9 tracrRNA, ATTO™ 550 (iDT). We designed a dsDNA donor template to include the desired correction along with silent mutations for screening purposes (novel restriction site for Bsh1236i, CG^CG). We generated the dsDNA donor template by overlapping PCR of two primers (Sigma, Table 1).

**Table 1:** gRNA and oligos used for off-target screening and dsDNA donor template production.

		Name	Location	MM	Sequence	PAM	On target/off target scores (CRISPOR)	
gRNA		gRNA_OAT	chr10:124398058	0	ACTTCGAGATAATGGACCTC	TGG	57/91	
Off targets	Region	Name	Location	MM	Sequence	PAM	Primer sequence (5′-3′)	
ON TARGET	exon	T > C L402P	chr10:124398058	0	ACTTCGAGATAATGGACCTC	TGG	Fw	CCGCCTCCTGGTTCAAGCGATTC
							Rv	CATGGGAGTGGAATGTGCCC
OFT1	exon/pseudogene	RP11-344 N17.12 *OAT pseudogene*	chX:48239690	1	ACTTCAAGATAATGGACTTC	CTT	Fw	CTGATGATGTCGCCATGGGT
							Rv	ATCCACTAGGCTGCCAAGTG
OFT2	intron	STS	chX:7255114	4	TTATCGAGATAAAGGACCTC	AGG	Fw	GGCCCCGGGATAAAATTTGC
							Rv	TGGCAAGCTCCTACCAAAGT
OFT3	intergenic	BTF3L4P4|PALLD	ch4:169534839	4	AATTAGAAATAATGAACCTC	TGG	Fw	CTCCTTTGGCTGGTTTGTGC
							Rv	GACAGGAGGCAGGAATCCAG
OFT4	exon	MUC5AC	chr11:1215504	4	GAGGTCCATCATCCTGGAGT	ACC	Fw	GGGACAGCAGGAAGGACTTC
							Rv	CCCCACCTCGTTTGTCATCA
OFT5	exon	RP11-133 K1.2/PAK6	ch15:40564866	4	CCGTCAAGATGATGGACCTC	AGG	Fw	CCCAACTCCTCTTTCCGACC
							Rv	GTCCTCCCACCTCGTTGAAG
OFT6	intergenic	FZD10|RP11-143E21.1	chr12:104109505	4	AGTTGGAGAGAATGGACCTG	GAG	Fw	TGGCATTGTCCAGCCCAGCTT
							Rv	AGTGTATGGCAGCAACTGACCG
DNA donor template		Fw	AAGGGATGTTTAAACGTTTATCTTTGAGCATGTACGTTTTACTATTTTTCTTTAGATTGGGATGCTTGGAAGGTGTGTCTACGACTTCGCGATAACGGGCTGCTCGCCAA					
		Rv	GTCTTGTTAATAATTTCAATGGACTCTCGAAGCTCATCCTCCTTGATCACCAGCGGAGGCGCAAACCTGATAATGTCGCCATGGGTTGGCTTGGCGAGCAGCCCGTTATC					

### 2.5 Genetic correction of c.1205 T > C in mutant iPSC

For each electroporation experiment, we dissociated 2 × 10^6 patient-derived iPSC to single cells with StemPro Accutase (ThermoFisher Scientific). We electroporated the sgAlt-R® S.p. HiFi Cas9 Nuclease V3, Alt-R® Cas9 electroporation enhancer (all from integrated DNA Technologies – iDT), sgRNA, and dsDNA donor template into the cells with Neon transfection systems ((ThermoFisher, 1100 V, 20 ms, 2 pulses). Cells were plated onto Matrigel-coated plates containing E8 with 5 μM ROCK inhibitor (Y-27632, Selleckchem) and 15 μM Alt-R® HDR Enhancer (iDT), and incubated overnight at 37 °C, 5% CO2.

24–48 h after electroporation, we sorted ATTO550 positive cells for monoclonal expansion in E8 with 5 μM ROCK inhibitor, 1% penicillin-streptomycin and 10% CloneR (StemCell Technologies). The medium was refreshed every 72 h until splitting. We individually screened a total of 55 monoclonal colonies by enzymatic digestion (Bsh1236i). All the clones were validated by Sanger sequencing (Eurofins Genomics).

### 2.6 Off-target analysis

We selected and designed PCR primers (Table 1, Sigma) for the top-6 off-target sites based on the off-target score for the given gRNA, as found in the online tools https://benchling.com and CRISPOR. Sequences were confirmed by Sanger sequencing of the PCR products (see Table 1 for primers).

### 2.7 Ornithine measurement in cell media

We measured Ornithine concentration in iPSC media (n = 4/cell line) using Ornithine Assay Kit (abcam, BioVision). Results are expressed in μM. We based the statistical analysis on Tukey test for multiple comparison of group means.

### 2.8 Targeted metabolomic analysis

We cultured the original (c.1205C/C) and edited (c.1205 T/T) iPSC lines (passage 15–20) to 60–95% confluency and collected 4 replicates of each line in 400 μl of extraction solvent (ACN:MeOH:MQ; 40:40:20). For targeted analysis of ornithine-related metabolites, FIMM metabolomics unit sonicated and analyzed the soluble fraction with Thermo Vanquish UHPLC+ system, coupled to a Q Exactive Orbitrap quadrupole mass spectrometer (ThermoFisher Scientific). Gradient on a SeQuant ZIC-pHILIC (2.1 × 100 mm, 5-μm particle) column (Merck) was used with Mobile phase A (20 mM ammonium carbonate, pH 9.4), and B (acetonitrile). Instrument control operated with the Xcalibur 4.1.31.9 software (ThermoFisher Scientific).

Metabolite peaks were integrated with TraceFinder™ 5.1 software using retention times confirmed with standards (inhouse library and library kit MSMLS-1EA, Merck). Data quality was monitored with pooled human serum as Quality Control (QC). We ran post-measurement analysis with data normalized to total protein (Pierce BCA Protein Assay Kit, ThermoFisher) within each prepared sample. Volcano plots generated in Rstudio with the ‘EnhancedVolcano’ package. Results shown as a matrix of fold change (cut at 0.5) *versus* p.value (cut at 0.05).

## 3 Results

### 3.1 Genetic correction of disease mutation in patient iPSC

**Fig. 1:**
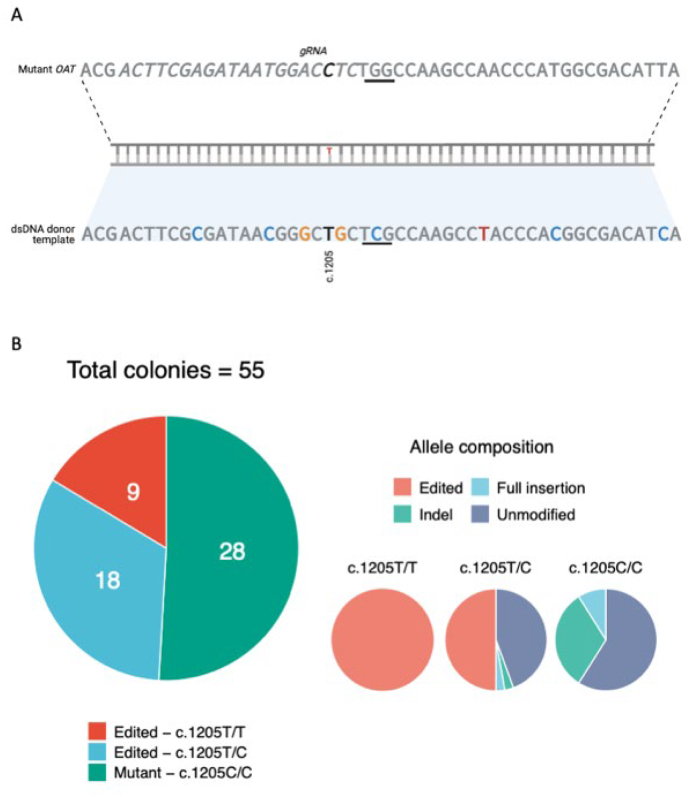
Efficiency of gene editing approach. A Sequence of mutant *OAT* gene and of the dsDNA donor template used for correction of the mutation. C.1205 base showed in black, gRNA sequence marked in *italics*, promoter adjacent motif (PAM) *underlined*, and silent modifications for screening in colors. B Editing efficiency in monoclonal colonies by genotype (right). Breakdown of allele composition (left), where ‘Indels’ refers to short insertion or deletions while ‘Full insertion’ refers to the insertion of the whole donor template in the *OAT* sequence.

We established HOGA_Fin_ iPSC lines and subsequently corrected the HOGA_Fin_ mutation with our selected CRISPR/Cas9 design (Fig. 1A). We obtained 55 monoclonal colonies. Our editing approach, comprising the homologous recombination of a double-strand DNA (dsDNA) donor template, showed a recombination efficiency close to 49% (Fig. 1B). It generated a total of 27 edited clones, 18 with one and 9 with both alleles corrected (heterozygotes and homozygotes, respectively).

We detected and excluded from further analysis 2 heterozygotes and 19 non-corrected clones with unintentional alterations (indels) in the targeted region.

Nonetheless, the analysis of the top six predicted off-target regions did not reveal off-target modifications in any of the edited iPSC lines (Table 1).

### 3.2 Characterization of iPSC lines

To ensure the quality and function of all our iPSCs, we tested their genetic integrity and pluripotency ability (see Supplementary data). A PCR for EBNA1 and OriP in 8 original patient cell lines confirmed that our iPSC lines did not retain the vectors employed for reprogramming (data not shown). All iPSC lines, before and after correction, presented distinctive hiPSC morphology and positive immunostainings of pluripotency markers (Nanog, Lin28, Tra-1-60, Supplementary Fig. 1). Furthermore, quantitative measurement by qPCR of pluripotency markers OCT4, Nanog and Sox2 in corrected cell lines revealed expected expression patterns, indicating that the editing process did not affect these characteristic pluripotency genes (data not shown). During embryoid body differentiation, all cell lines successfully differentiated into the three germ layers (Supplementary Fig. 1). Importantly, neither the reprogramming nor the editing process affected the karyotypes of edited or non-edited cell lines, as shown by G-banding of iPSCs from both patients (Supplementary Fig. 2). Along with the genetic results, these analyses suggest that the editing process did not impair neither the function nor characteristics of our iPSCs.

### 3.3 Analysis of protein expression

**Fig. 2:**
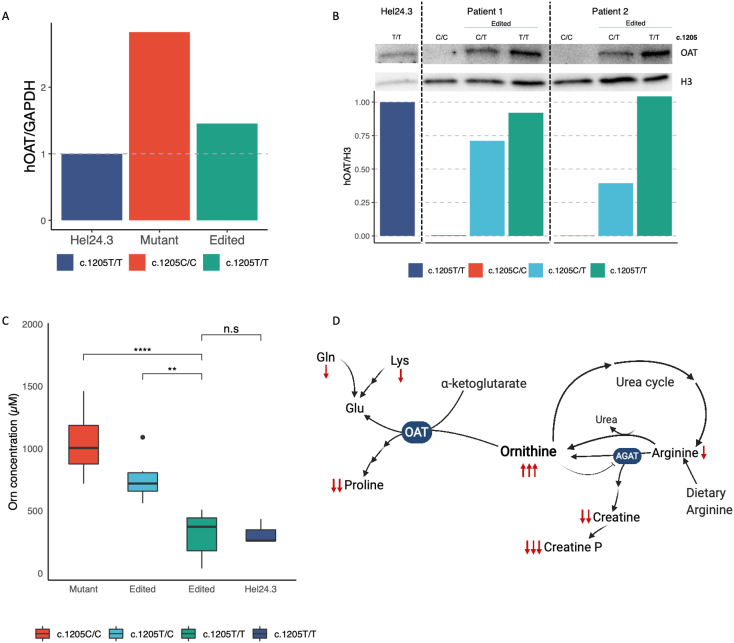
Metabolic phenotypic characterization of iPSC lines. A hOAT mRNA expression levels, relative to GAPDH, in control (Hel24.3), mutant and homozygous-edited iPSC. B Western blot and quantification of relative protein levels using histone H3 as reference. C Ornithine concentration in cell media of mutant (C/C), edited (heterozygotes, C/T: homozygotes T/T), and control (T/T) iPSC lines (n = 4/cell line). Significance based on Tukey test (p-value codes: 0.0001 ‘****’; <0.01 ‘**’; <0.05 ‘*’; >0.05‘n.s’). D Ornithine related pathways showing affected metabolites in patient cells. ↑↑↑ highly increased; ↓ slightly decreased, ↓↓ moderately decreased, ↓↓↓ highly decreased. Created with BioRender.com.

The predominant Finnish HOGA-causing mutation c.1205 T > C p.(Leu402Pro) likely destabilizes the protein structure, ultimately leading to its degradation, as seen in patient fibroblasts [7]. Accordingly, we observed comparable mRNA expression in Hel24.3 (stable iPS cell line used as a control, c.1205 T/T), mutant iPSC, and iPSC edited in homozygosity by qPCR. We did not detect, however, OAT protein in our iPSC derived from homozygous HOGA_Fin_ patients. After the genetic correction, the iPSC lines edited in hetero- and homozygosity showed a strong production of OAT in the Western Blot analysis (Fig. 2A).

### 3.4 Restoration of metabolic pathways

Given that patients present with elevated ornithine in circulation and body fluids, we measured ornithine concentration in cell media to assess OAT function in our cell model. Media from all corrected cell lines presented ornithine levels comparable to those in media from control iPSC lines (Hel24.3), significantly lower than those of the homozygous-mutant HOGA iPSCs (Fig. 2B). Our results indicate that the correction of the *OAT* mutation leads to the recovery of the enzymatic activity.

**Fig. 3:**
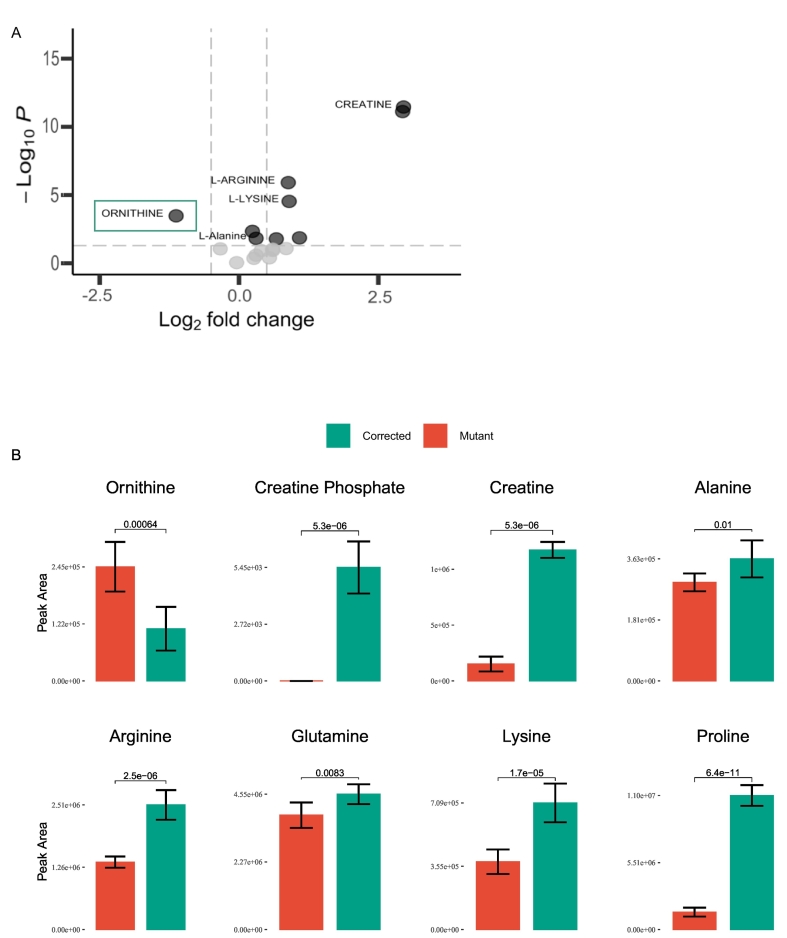
A. Volcano plot analysis of metabolic changes in corrected patient-derived iPSC (c.1205T/T) relative to nonedited cell lines (fold change cut at 0.5; p.value cut at 0.05, n = 4/cell line). B. Breakdown of significantly changed metabolites levels in mutant versus corrected cell lines (n = 4/cell line).

OAT dysfunction triggers an intracellular imbalance of ornithine related metabolites (Fig. 2C). To evaluate the effects of protein function recovery, we explored these metabolic pathways in edited *versus* non-edited patient lines, using liquid chromatograph-mass spectrometry (LC-MS targeted metabolomics). We discovered that the levels of intracellular ornithine significantly decrease in edited cell lines when compared to the mutants (Fig. 3A-B). In addition, we found a significant increase in the intracellular levels of creatine, creatine phosphate, alanine, arginine, lysine, glutamine, and proline (Fig. 3A-B).

## 4 Discussion

Our results show the restoration of OAT enzymatic function after genetic correction in a novel iPSC model for HOGA_Fin_. Patients with HOGA present with complex metabolic imbalance, related to the hyperornithinemia caused by OAT dysfunction. Here, we investigated these intracellular metabolic changes by comparing the *OAT*-mutant and homozygously corrected cells. We observed restoration of metabolic homeostasis upon recovery of OAT activity, including increased levels of lysine, glutamate, glutamine, creatine and creatine phosphate reported depleted in patients [3,4,21].

While the mechanisms behind chorio-retinal degeneration remain elusive, type-II muscle fiber atrophy is thought to arise from the failure to generate enough creatine and creatine phosphate. The ornithine-mediated inhibition of L-arginine:glycine amidinotransferase (AGAT) plays a key role in the pathology [21]. AGAT produces guanidinoacetate, the precursor of creatine, from arginine. In line with these previous reports, we could show substantially reduced creatine and creatine phosphate concentration in the *OAT*-mutant cell lines. This emphasizes the importance of creatine supplementation usually included in the dietary treatment [26]^.^ Additionally, HOGA patients present with low plasma concentrations of lysine [27], and oral lysine treatment has lowered plasma ornithine levels [28] by the competition of lysine and ornithine in the tubular reabsorption of amino acids. Interestingly, we observed lower intracellular concentration of lysine in the *OAT*-mutant iPSC lines.

As part of the molecular characterization, we analyzed the quantitative *OAT* expression levels and OAT production. Our results show that mutant iPSC express *OAT* at the mRNA level but fail to produce the protein. This suggests that the Finnish-founder mutation under study, OAT c.1205 T > C p.(Leu402Pro), might destabilize the structure of the protein, ultimately leading to its degradation.

The current treatment strategies usually improve the muscular prognosis but only decelerate the progression of the retinal symptoms. Although long-term therapeutic alternatives have been proposed [29,30], neither enzyme nor gene replacement therapies exist to date.

Here, we sought to explore the genetic correction of the mutation directly in patient-derived iPSC. This approach allows the production of functional, autologous cells with the *OAT* expression under its physiological genetic control. Our iPSC feature lowered intracellular ornithine levels compared to their non-edited counterparts. The cell culture media of all our edited cell lines showed decreased ornithine concentration, albeit less pronounced in heterozygotes. This accords with previous observations of reduced OAT activity in carriers, although they show no clinical symptoms [31,32].

Importantly, the edition did not produce any detectable off-target modifications, neither did it affect the pluripotent quality of the iPSCs nor their chromosomal integrity. However, the occurrence of undesired, on-target alterations makes it currently necessary to obtain monoclonal lines, as the effects of these changes on the phenotype remain uncertain. These events challenge the immediate utilization of the presented genome editing approach as an *in vivo* therapeutic alternative. Nevertheless, we believe that our results offer new opportunities for the modelling of this disease, mechanistic studies, and the evaluation of novel therapies for gyrate atrophy. We predict that the constant advancement of CRISPR/Cas9 editing, and delivery systems will bring promising optimization strategies to make new therapeutic alternatives a reality.

## Funding

The work was funded by 10.13039/501100002341Academy of Finland (Grant 308481), Foundation for Paediatric Research, Sigrid Juselius Foundation, 10.13039/501100007417Paulo Foundation, Mary & Georg C. Ehrnrooth Foundation and 10.13039/501100003125Finnish Cultural Foundation. FIMM metabolomics unit was supported by HiLIFE and 10.13039/501100013840Biocenter Finland.

## Declaration of Competing Interest

The authors declare no conflict of interest.

## Appendix A Supplementary data

Supplementary material Image 1
