# Supplementary material for: CRISPR correction of the Finnish ornithine delta-aminotransferase mutation restores metabolic homeostasis in iPSC from patients with gyrate atrophy
Source: Mol Genet Metab Rep. 2022 Apr 1;31:100863. doi: 10.1016/j.ymgmr.2022.100863 (PMC9248217; doi:10.1016/j.ymgmr.2022.100863)
Supplement: Supplementary file 1 — Supplementary material [file mmc1.docx]

1. **Supplementary data**
   1. **Materials and methods**

**8.1.1 Cell culture**

We cultured human dermal fibroblasts in Dulbecco’s modified Eagle’s medium (DMEM) containing 10% fetal bovine serum (FBS), 100 µg/ml penicillin-streptomycin and 2 mM GlutaMAX (all ThermoFisher Scientific). Human iPSCs were cultured on Matrigel (Corning) coated plates in E8 medium (Gibco) and split using 0.5 mM EDTA (Sigma). We kept all cell lines at 37 °C and 5% CO2, with media change every other day. All cell lines tested negative for mycoplasma.

**8.1.2 Fibroblast culture**

Upon written informed consent, 2-3 mm forearm epidermal samples were taken under local anesthesia. The skin samples were mechanically dissociated and grown in DMEM supplemented with 20% FBS and 100 µg/ml penicillin-streptomycin (all ThermoFisher Scientific) until fibroblasts were confluent. Thereafter, fibroblasts were trypsinized using TrypLE Select (ThermoFisher) and split for expansion and freezing.

**8.1.3 Embryoid body differentiation**

To test the pluripotency for embryonal lineage differentiation, we grew the cells until 90-100% confluency and performed embryoid body (EB) differentiation as previously described [23]. Final plated EBs were fixed with 4%PFA-PBS (ThermoFisher) and immunostained for characteristic markers of the three germ-layers (Sox17, SMA, TUB-B3).

**8.1.4 Immunocytochemistry**

We fixed the cells with 4%PFA-PBS (ThermoFisher) and treated them with Ultra Vision block (ThermoFisher). For immunostaining, we diluted the primary antibodies in 0.1% Tween-20 PBS and incubated overnight at 4 °C with the given dilutions. After washing, we incubated at RT for 1 h with secondary antibody in the presence of Hoechst33342 (ThermoFisher) to stain the nuclei. Pictures were taken with EVOS (ThermoFisher) or Zeiss ApoTome2 (Zeiss) cell imaging systems.

Primary antibodies: NANOG (1: 500, D73G4, Cell Signaling), Lin28 (1:500, D84C11, Cell signaling), TRA-1-60 (MA1-023, ThermoFisher) TUB-B3 (1:500, MAB1195, R&D Systems), SMA (1:500, A2547, Sigma), SOX17 (1:500, AF1924, R&D Systems). Secondary antibodies: AlexaFluor 488: donkey anti-goat (1:500, A11055; Invitrogen), donkey anti-mouse (1:500, A21202 and A21203; Invitrogen) and donkey anti-rabbit (1:500, A21206 and A21207; Invitrogen).

**8.1.5 qPCR and Western Blot**

We analyzed quantitively expression levels of human OAT, as well as of pluripotency markers Nanog, OCT4 and Sox2 in iPSC lines by qPCR (primers from Sigma). We measured protein concentration (Pierce BCA Protein Assay Kit, ThermoFisher) and assessed the production of the OAT protein by Western blot (15µg per sample, Bio-Rad MiniPROTEAN TGX gel), probing the PVDF membranes (iBlot™ Transfer Stack, Invitrogen) with a polyclonal primary antibody against OAT (1:3500, PA5-27968, ThermoScientific). For detection, we used an anti-rabbit-HRP-coupled secondary antibody (1:5000, 7074S, Cell Signalling). For quantification, we probed the membrane with a rabbit polyclonal anti-H3 primary antibody (ab47915, Abcam) to obtain relative protein values.

Primers:

| hOAT_Fw | AGACTGCCTGTAAACTAGCTCGTAAG |
| --- | --- |
| hOAT_Rv | ACTGGAGATAGCAGACAACGTCCT |
| hOCT4_Fw | TTGGGCTCGAGAAGGATGTG |
| hOCT4_Rv | TGCATAGTCGCTGCTTGATC |
| hSOX2_Fw | GCCCTGCAGTACAACTCCAT |
| hSOX2_Rv | TGCCCTGCTGCGAGTAGG |
| hNanog_Fw | CTCAGCCTCCAGCAGATGC |
| hNanog_Rv | TAGATTTCATTCTCTGGTTCTGG |
| GAPDH_Fw | AGAAGGCTGGGGCTCATTTG |
| GAPDH_Rv | AGGGGCCATCCACAGTCTTC |

**8.1.6 Karyotype analysis**

We treated edited (n=3/patient) and non-edited (n=2/patient) iPSC (passage 10-20) with colcemid for 4 h, 37°C, 5% CO2. We dissociated with TrypLE Select, collected them with DMEM (both ThermoFisher) and centrifuge 200 xg, 5 min. We remove most of the supernatant (SN) and resuspend the cells. We incubated 10 min, 37°C with 3ml prewarmed KCl, followed by centrifugation 200 xg 5min. We removed most of the SN and resuspended the cells. We fixed the cells by washing twice with 3 mL fixing solution (1;3 acetic acid;methanol) and centrifuging 200 xg 5 min. After the last centrifugation, we added the final 3 mL and sent the samples to Ambar Anàlisis Mèdiques (Barcelona, Spain) for karyotyping by G-banding.

- 1. **Supplementary Figures**

**Fig. 1. iPSC characterization and embryoid body differentiation.** Immunostaining against pluripotency markers (Nanog, Tra-1-60 and Lin28) and characteristic markers of the three germ layers: ectoderm (Tub-B3); mesoderm (smooth muscle actin – SMA); endoderm (Sox17).

**Fig. 2. Karyotype analysis by G-banding.** Mutant and edited cell lines preserved their chromosomal integrity after reprogramming and genome editing (passage 10-20).
